# Cancer-related risk factors and incidence of major cancers by race, gender and region; analysis of the NIH-AARP diet and health study

**DOI:** 10.1186/s12885-017-3557-1

**Published:** 2017-08-30

**Authors:** Tomi Akinyemiju, Howard Wiener, Maria Pisu

**Affiliations:** 1 Department of Epidemiology, University of Alabama at Birmingham, Birmingham, AL USA; 2 Comprehensive Cancer Center, University of Alabama at Birmingham, Birmingham, AL USA; 3 Department of Epidemiology, University of Kentucky, Lexington, KY 40504 USA; 4 Division of Preventive Medicine, University of Alabama at Birmingham, Birmingham, AL USA

**Keywords:** Cancer-related risk factors, Cancer incidence, Obesity, Diet, Physical activity

## Abstract

**Background:**

Racial disparities in the incidence of major cancers may be attributed to differences in the prevalence of established, modifiable risk factors such as obesity, smoking, physical activity and diet.

**Methods:**

Data from a prospective cohort of 566,398 adults aged 50–71 years, 19,677 African-American and 450,623 Whites, was analyzed. Baseline data on cancer-related risk factors such as smoking, alcohol, physical activity and dietary patterns were used to create an individual adherence score. Differences in adherence by race, gender and geographic region were assessed using descriptive statistics, and Cox proportional hazards models were used to determine the association between adherence and cancer incidence.

**Results:**

Only 1.5% of study participants were adherent to all five cancer-related risk factor guidelines, with marked race-, gender- and regional differences in adherence overall. Compared with participants who were fully adherent to all five cancer risk factor criteria, those adherent to one or less had a 76% increased risk of any cancer incidence (HR: 1.76, 95% CI: 1.70 – 1.82), 38% increased risk of breast cancer (HR: 1.38, 95% CI: 1.25 – 1.52), and doubled the risk of colorectal cancer (HR: 2.06, 95% CI: 1.84 – 2.29). However, risk of prostate cancer was lower among participants adherent to one or less compared with those who were fully adherent (HR: 0.79, 95% CI: 0.75 – 0.85). The proportion of cancer incident cases attributable to low adherence was higher among African-Americans compared with Whites for all cancers (21% vs. 19%), and highest for colorectal cancer (25%) regardless of race.

**Conclusion:**

Racial differences in the proportion of cancer incidence attributable to low adherence suggests unique opportunities for targeted cancer prevention strategies that may help eliminate racial disparities in cancer burden among older US adults.

**Electronic supplementary material:**

The online version of this article (doi:10.1186/s12885-017-3557-1) contains supplementary material, which is available to authorized users.

## Background

Colorectal, prostate and breast cancer are three of the four most common cancers among adults in the U.S. Combined, they are estimated to account for over 560,000 new cases and 115,000 deaths due to cancer in 2016 [1]. Advances in our understanding of risk factors, screening techniques and cancer treatment have led to significant declines in incidence and mortality over the past several decades. However, African-Americans remain at disproportionately higher risk of developing prostate [2] and colorectal [3] cancers, and when diagnosed tend to have highly aggressive cancer phenotypes compared with whites [4, 5]. The fundamental cause of disparities in cancer incidence has been the subject of vigorous investigations for many years, however these racial differences have persisted. Differences in racially, socio-economically and geographically patterned etiologic risk factors [6–8] such as obesity (48% in African-American versus 33% in Whites) [9] and physical inactivity (61% in African-American versus 45% in Whites) [10], have emerged as potentially modifiable risk factors that may contribute the observed disparities in cancer outcomes in US adults. Importantly, recent studies estimate that up to 50% of all new breast cancer cases could be prevented through healthy behaviors, specifically body weight, physical activity, alcohol intake and smoking [11]. These are also critical risk factors for colorectal [12, 13] and prostate [14, 15] cancers.

In this prospective cohort of African-American and White older adults, we examined adherence to body weight, physical activity, alcohol, smoking and nutrition guidelines by race, gender and region, and estimated the proportion of overall, breast, prostate and colorectal cancer incidence attributable to poor adherence. Understanding the contribution of these modifiable risk factors to cancer incidence may be useful for public health interventions focused on cancer prevention and inform strategies to eliminate racial and/or geographic disparities in cancer risk.

## Methods

### Study participants

Data for this study was obtained from the prospective National Institutes of Health-American Association of Retired Persons (NIH-AARP) Diet and Health Study. The cohort consists of 566,398 adults AARP members aged 50–71 years recruited in 1995–1996 (Additional file 1: Figure S1). At enrollment, participants completed a baseline questionnaire assessing lifestyle and behavioral risk factors such as smoking, alcohol, physical activity and dietary patterns. Participants with self-reported cancer at baseline (*n* = 49,318), proxy respondents (*n* = 15,760), death record data only (*n* = 4255) or who had missing data on behavioral risk factors (40,676) and race (9566) were excluded from analysis. The final analysis included a total of 470,000 adults; 19,677 African-American and 450,623 Whites with no prior history of any cancer. With a sample size of 19,677 for African-Americans, we were well powered with Type 1 error of 0.05 and Type II error of 80% to detect effect sizes as low as 1.1 and adherence levels as low as 20%.

### Ascertainment of cancer incidence

Incident cancer cases were identified through a linkage to state cancer registries through December 31, 2012. Detailed information for each cancer diagnosis was obtained on diagnosis date, stage, grade, and first course of treatment within the first year of diagnosis. Incident cancer ascertainment has been estimated to be about 90% complete [16].

### Cancer-related risk factors

The American Cancer Society (ACS) [17] and the World Cancer Research Fund/American Institute for Cancer Research (WCRF/AICR) [18] developed specific guidelines regarding body weight, physical activity, diet, smoking and alcohol consumption to guide cancer prevention efforts. Here, we assessed adherence to the WCRF/AICR guidelines on five cancer-related risk factors; physical activity, body weight, alcohol use, smoking and nutrition (fruit and vegetable intake). We used self-reported measures obtained during enrollment based on the 12-month period prior to enrollment. Each participant was assigned a score of 1 if fully adherent, 0.5 if partially adherent, and 0 if not adherent (Table 1). Each risk factor was weighted equally and adherence scores were summed up to create a total adherence score ranging from 0 to 5.

**Table 1:** Cancer related risk factors adherence criteria

Risk Factor	Adherence Guideline	Adherence Score
Physical Activity (# of 20 min activities)	≥5 per week	1
	≥1 per month - < 5 per week	0.5
	<1 per month	0
Obesity (BMI)	≥18.5 - ≤ 25 kg/m^2^	1
	>25 - ≤ 30 kg/m^2^	0.5
	<18.5 or >30 kg/m^2^	0
Alcohol Use (# drinks per week)	Women ≤7, Men ≤14	1
	Women >7 - ≤ 14, Men >14 ≤ 28	0.5
	Women >14, Men >28	0
Nutrition (Fruit and Vegetable Servings per day)	≥5	1
	≥3 - < 5	0.5
	<3	0

### Statistical analysis

We assessed adherence to each cancer prevention guideline overall (by summing the total adherence score) and for each risk factor separately. We compared baseline characteristics and adherence by race and gender using chi-square tests and ANOVA as appropriate. We also examined differences in adherence by geographic region, categorized as: Northeast, Mid-West, South, and West. We conducted Cox proportional hazards models to determine the association between adherence and cancer incidence, and reported the results from Cox models as hazard ratios (HR) and 95% confidence intervals. We examined Kaplan-Meier survival cures and found no evidence of violations of the proportional hazards assumption. All statistical models were stratified by race, and adjusted for baseline characteristics such as age, marital status, education, health status, and gender (for colorectal cancer). Trend tests were performed by assessing the linear relationship between adherence and cancer incidence. Censoring occurred at the time of first primary cancer diagnosis, loss to follow up or the end of incidence follow-up period, whichever occurred first. The attributable risk (AR) due to adherence was calculated from models based on individual’s region, race, background covariates, and adherence value using the appropriate model, and the counter-factual estimate for that individual assuming the highest rate of adherence. The proportions of individuals categorized as affected (i.e. for which the random number did not exceed the risk estimate) for both situations (i.e. factual and counter factual) were divided to form a risk ratio (RR), and AR calculated using the formula (RR-1)/RR. Confidence intervals for the AR were generated from bootstrapped resamples of 1000 draws of random numbers from a uniform distribution and compared to the estimates, and this was repeated for the counterfactual estimates to provide a measure of the precision of AR estimates. All analyses were conducted using SAS 9.4 and R statistical package.

## Results

### Characteristics of study population

The majority of NIH-AARP participants were between ages 65 to 69 years (32%), and most participants were male (60%), married (69%) and 39% had at least a college degree (Table 2). About 69% of participants rated their health status as good or very good. The median follow-up time was 15.5 person-years (Std. Dev: 4.8) for both African-Americans and Whites.

**Table 2:** Baseline Characteristics of NIH-AARP Study Participants, 1995-1996

	Overall	White	AA
Age Category			
< 55 years	64,491 (13.71%)	61,318 (13.61%)	3173 (16.13%)
55-59 years	106,893 (22.73%)	101,588 (22.54%)	5305 (26.96%)
60-64 years	132,005 (28.07%)	126,108 (27.99%)	5897 (29.97%)
65-69 years	150,255 (31.95%)	145,423 (32.27%)	4832 (24.56%)
> =70 years	16,656 (3.54%)	16,186 (3.59%)	470 (2.39%)
Gender			
Male	280,558 (59.66%)	272,444 (60.46%)	8114 (41.24%)
Female	189,742 (40.34%)	178,179 (39.54%)	11,563 (58.76%)
Marital Status			
Married	323,303 (69.11%)	314,122 (70.05%)	9181 (47.27%)
Widowed	51,660 (11.04%)	48,293 (10.77%)	3367 (17.34%)
Divorced	64,882 (13.87%)	60,310 (13.45%)	4572 (23.54%)
Separated	5483 (1.17%)	4445 (0.99%)	1038 (5.34%)
Never Married	22,508 (4.81%)	21,244 (4.74%)	1264 (6.51%)
Education			
< 8 years	27,821 (6.07%)	25,646 (5.83%)	2175 (11.66%)
8-11 years	93,358 (20.37%)	89,446 (20.35%)	3912 (20.98%)
12 years/High School	46,651 (10.18%)	44,926 (10.22%)	1725 (9.25%)
Post-High School/Some College	109,302 (23.85%)	104,369 (23.74%)	4933 (26.46%)
College or post-grad	181,132 (39.53%)	175,231 (39.86%)	5901 (31.65%)
Health Status			
Excellent	81,207 (17.50%)	79,438 (17.86%)	1769 (9.20%)
Very good	166,103 (35.80%)	160,658 (36.13%)	5445 (28.31%)
Good	160,182 (34.53%)	152,225 (34.23%)	7957 (41.37%)
Fair	48,823 (10.52%)	45,256 (10.18%)	3567 (18.55%)
Poor	7641 (1.65%)	7145 (1.61%)	496 (2.58%)
State of Residence			
CA	139,633 (29.69%)	135,081 (29.98%)	4552 (23.13%)
FL	100,509 (21.37%)	98,147 (21.78%)	2362 (12.00%)
GA	13,663 (2.91%)	12,468 (2.77%)	1195 (6.07%)
LA	18,225 (3.88%)	16,901 (3.75%)	1324 (6.73%)
MI	24,420 (5.19%)	22,254 (4.94%)	2166 (11.01%)
NC	39,889 (8.48%)	37,678 (8.36%)	2211 (11.24%)
NJ	60,484 (12.86%)	57,755 (12.82%)	2729 (13.87%)
PA	73,477 (15.62%)	70,339 (15.61%)	3138 (15.95%)
Cancer Type			
Any Cancer	114,392 (24.33%)	109,971 (23.99%)	4421 (22.47%)
Breast Cancer	12,698 (6.70%)	12,020 (6.75%)	678 (5,87%)
Prostate Cancer	30,664 (10.93%)	29,222 (10.73%)	1442 (17.77%)
Colorectal Cancer	10,300 (2.19%)	9845 (2.19%)	455 (2.31%)

For breast and prostate cancer, the percentages in the above table are based on females only and males only, respectively

### Adherence to cancer-related risk factors

**Fig. 1:**
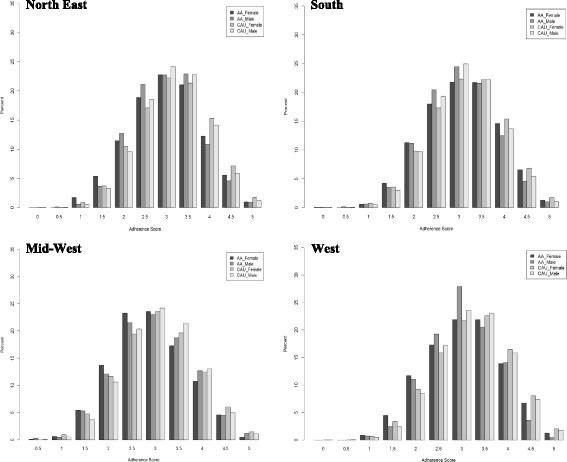
Distribution of adherence components by race and gender, stratified by region, NIH-AARP Diet and Health Study

Only 1.5% of study participants were adherent to all five cancer-related risk factor guidelines, with marked race-, gender- and regional differences in adherence overall (Fig. 1). Adherence to each risk factor guideline also varied significantly by gender and region (Table 3). ***Obesity***: Only 35% of participants met the adherence criteria for obesity or body weight (defined as BMI between 18.5 and 25), 22% did not meet the criteria at all, and 43% were overweight. ***Alcohol Use*** **:** Adherence to guidelines regarding alcohol was high, with over 98% of participants meeting the criteria i.e. consuming 7 or less alcoholic drinks per week for females and 14 or less alcoholic drinks per week for males. ***Smoking***: Less than 40% of participants were adherent to guidelines regarding smoking i.e. never smokers, while 52% were partially adherent meaning that they were former but not current smokers. ***Nutrition***: Only 26% of study participants were adherent to nutrition guidelines, and 36.5% were totally non-adherent i.e. did not consume at least 5 servings of fruits and vegetables per day. ***Physical Activity***: Only 23% of study participants were adherent to physical activity guidelines i.e. at least 210 min of moderate physical activity per week.

**Table 3:** Adherence to Specific Cancer Risk Factors by Race, Gender and Region, NIH-AARP Diet and Health Study (%^a^)

	Obesity			Alcohol			Smoking			Nutrition			Physical Activity		
	0	0.5	1	0	0.5	1	0	0.5	1	0	0.5	1	0	0.5	1
Overall	21.71	42.96	35.33	0.29	0.85	98.85	11.89	51.82	36.29	36.54	37.75	25.71	20.57	56.81	22.61
Gender															
Male	20.47	49.87	29.66	0.40	1.15	98.45	10.37	59.29	30.34	35.40	38.17	26.43	16.91	58.25	24.84
Female	23.59	32.49	43.92	0.13	0.40	99.47	14.20	40.49	45.31	38.26	37.13	24.61	26.13	54.63	19.24
Race															
White	21.21	42.96	35.83	0.29	0.85	98.86	11.74	52.10	36.15	36.73	38.07	25.20	20.27	56.95	22.78
AA	34.27	42.99	22.74	0.34	0.92	98.74	15.58	44.74	39.68	31.85	29.87	38.28	28.10	53.37	18.53
Region															
Midwest	26.46	42.98	30.55	0.35	0.79	98.87	13.19	50.95	35.86	36.91	37.37	25.72	24.21	56.97	18.81
North East	23.58	44.34	32.09	0.26	0.76	98.97	11.69	50.45	37.86	33.38	38.61	28.01	23.70	56.48	19.83
South	20.95	43.20	35.85	0.33	0.96	98.71	12.92	52.97	34.11	38.96	36.90	24.15	19.58	57.01	23.41
West	20.11	41.38	38.51	0.29	0.89	98.83	10.60	51.83	37.56	36.46	38.07	25.47	18.27	56.86	24.87
Race-Gender															
White Males	20.27	49.87	29.85	0.40	1.14	98.46	10.23	59.41	30.36	35.44	38.38	26.18	16.73	58.31	24.96
White Females	22.66	32.16	45.18	0.12	0.40	99.48	14.12	40.68	45.21	38.74	37.59	23.67	25.81	54.82	19.37
AA Males	27.55	49.68	22.76	0.45	1.52	98.03	15.50	54.89	29.61	34.01	30.66	35.33	23.46	56.04	20.50
AA Females	39.22	38.06	22.72	0.25	0.48	99.27	15.63	37.26	47.10	30.27	29.29	40.44	31.52	51.39	17.09

Risk factors defined based on WCRF/AICR criteria for adherence; 0 if not met, 0.5 if partially met and 1.0 if fully met

^a^Proportion of study participants at each level of adherence

### Adherence to guidelines and cancer incidence

**Fig. 2:**
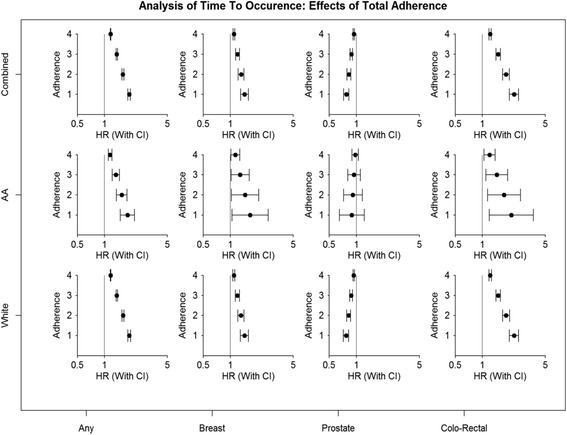
Multivariable adjusted hazard ratios (HR, 95% CI) for adherence and cancer incidence, stratified by race, NIH-AARP Diet and Health Study

Increasing adherence to cancer prevention guidelines was associated with progressively reduced risk of any cancer incidence (Table 4, Fig. 2). Compared with participants who were fully adherent to all five cancer risk factor criteria, those adherent to one or less had a 76% increased risk of cancer incidence (HR: 1.76, 95% CI: 1.70 – 1.82), those adherent to two criteria had a 53% increased risk (HR: 1.53, 95% CI: 1.49 – 1.56), and those adherent to four had a 15% increased risk (HR: 1.15, 95% CI: 1.14 – 1.16, p-trend <0.001). Similar associations were observed for Whites as well as African-Americans. Breast cancer incidence increased significantly with reduced overall adherence, with a 38% increased risk of breast cancer among participants adherent to one or no criteria (HR: 1.38, 95% CI: 1.25 – 1.52, p-trend <0.001). Similar magnitude of association was observed among Whites as well as African-Americans, although the results for African-Americans were not statistically significant. Prostate cancer incidence appeared to be inversely associated with adherence, with a 21% reduced risk among participants adherent to only one or no criteria (HR: 0.79, 95% CI: 0.75 – 0.85, p-trend <0.001) and a 6% reduced risk among those adherent to four criteria compared with five (HR: 0.94, 95% CI: 0.93 – 0.96, p-trend 0.001), although the association among African-Americans was not statistically significant. The risk of colorectal cancer increased by over 100% among participants adherent to one or no criteria (HR: 2.06, 95% CI: 1.84 – 2.29, p-trend <0.001) compared with those adherent to all five, and the association was non-significant among African-Americans. Adherence to one or none of criteria compared with all five was associated with over 100% increased risk of any cancer in the South (HR: 2.09, 95% CI: 1.83-2.38) and North-East (HR: 2.01, 95% CI: 1.86-2.17), and a 79% and 83% increased risk in the Mid-West and West respectively (Table 5).

**Table 4:** Association between Adherence and Any, Breast, Colorectal and Prostate Cancer Incidence by Race, NIH-AARP Diet and Health Study

Any Cancer HR (95% CI)				Breast Cancer HR (95% CI)			Prostate Cancer HR (95% CI)			Colorectal Cancer HR (95% CI)		
	All	White	AA	All	White	AA	All	White	AA	All	White	AA
Obesity												
0	1.08 (1.06 -1.1)	1.08 (1.06 - 1.1)	0.91 (0.83 - 0.99)	1.16 (1.1 - 1.22)	1.14 (1.09 - 1.2)	1.1 (0.89 - 1.37)	0.87 (0.84-0.89)	0.87 (0.84 -.89)	0.89 (0.76 - 1.03)	1.3 (1.22 - 1.38)	1.29 (1.2-1.37)	1.09 (0.83 - 1.43)
0.5	1.04 (1.03 -1.05)	1.04 (1.03 -1.05)	0.95 (0.91 - 0.99)	1.08 (1.05 - 1.1)	1.07 (1.04 -1.09)	1.05 (0.94 - 1.17)	0.93 (0.91-0.95)	0.93 (0.92 -0.95)	0.94 (0.87 - 1.02)	1.14 (1.11 -1.17)	1.14 (1.11 -1.17)	1.04 (0.91 - 1.2)
*P-trend*	*<0.001*	*<0.001*	*0.031*	*<0.001*	*<0.001*	*0.3713*	*<0.001*	*<0.001*	*0.1258*	*<0.001*	*<0.001*	*0.53*
Alcohol												
0	1.55 (1.43 -1.68)	1.54 (1.44 -1.66)	1.64 (1.16 - 2.31)	1.78 (1.26 - 2.51)	1.5 (1.07 - 2.09)	2.71 (1.06 - 6.9)	1.08 (0.92 -1.27)	1.04 (0.89 - 1.2)	1.05 (0.56 - 1.95)	2.02 (1.59 -2.58)	1.99 (1.59 -2.47)	3.64 (1.6 - 8.27)
0.5	1.24 (1.19 -1.29)	1.24 (1.2 -1.29)	1.28 (1.08 - 1.52)	1.33 (1.12 - 1.59)	1.22 (1.04 -1.45)	1.64 (1.03 - 2.63)	1.04 (0.96 -1.13)	1.02 (0.94 - 1.1)	1.02 (0.75 - 1.4)	1.42 (1.26 -1.61)	1.41 (1.26 -1.57)	1.91 (1.27 - 2.88)
*P-trend*	*<0.001*	*<0.001*	*<0.001*	*<0.001*	*0.01763*	*0.037*	*0.359*	*0.65*	*0.89*	*<0.001*	*<0.001*	*0.0036*
Smoking												
0	1.68 (1.65 -1.72)	1.7 (1.67 - 1.74)	1.63 (1.49 - 1.79)	1.11 (1.05 - 1.18)	1.12 (1.06 -1.18)	1.28 (1.02 - 1.6)	0.83 (0.79 -0.86	0.82 (0.79 - .86)	0.88 (0.75 - 1.05)	1.41 (1.32 -1.51)	1.42 (1.33 -1.51)	1.3 (0.97 - 1.73)
0.5	1.3 (1.28 -1.31)	1.31 (1.29 -1.32)	1.28 (1.22 - 1.34)	1.06 (1.03 - 1.09)	1.06 (1.03 -1.09)	1.13 (1.01 - 1.27)	0.91 (0.89-0.93)	0.91 (0.89 -0.93)	0.94 (0.86 - 1.02)	1.19 (1.15 -1.23)	1.19 (1.15 -1.23)	1.14 (0.99 -1.31)
*P-trend*	*0*	*0*	*<0.001*	*<0.001*	*<0.001*	*0.033*	*<0.001*	*<0.001*	*0.1589*	*<0.001*	*<0.001*	*0.07298*
Nutrition												
0	1.11 (1.09 -1.13)	1.11 (1.1 - 1.13)	1.09 (1.01 - 1.17)	1.07 (1.01 - 1.12)	1.07 (1.02 -1.12)	1.13 (0.94 - 1.37)	1.02 (0.99 -1.05)	1.02 (0.99 -1.05)	0.93 (0.82 - 1.06)	1.13 (1.07 -1.19)	1.13 (1.08 - 1.2)	1.15 (0.92 - 1.44)
0.5	1.05 (1.04 -1.06)	1.05 (1.05 -1.06)	1.04 (1.01 - 1.08)	1.03 (1.01 - 1.06)	1.04 (1.01 -1.06)	1.06 (0.97 - 1.17)	1.01 (0.99 -1.03)	1.01 (0.99 -1.03)	0.96 (0.91 - 1.03)	1.06 (1.03 -1.09)	1.07 (1.04 -1.09)	1.07 (0.95 - 1.2)
*P-trend*	*<0.001*	*<0.001*	*0.025*	*0.01495*	*0.004*	*0.199*	*0.254*	*0.174*	*0.2847*	*<0.001*	*<0.001*	*0.2305*
Physical Activity												
0	1.15 (1.13 -1.18)	1.15 (1.13 -1.17)	1.18 (1.07 - 1.3)	1.12 (1.05 - 1.19)	1.11 (1.05 -1.18)	1.28 (0.99 - 1.67)	0.94 (0.90-0.97)	0.92 (0.88 -0.95)	1.04 (0.87 - 1.24)	1.33 (1.24 -1.42)	1.35 (1.26 -1.44)	1.15 (0.85 -1.56)
0.5	1.07 (1.06 -1.09)	1.07 (1.06 -1.08)	1.09 (1.03 - 1.14)	1.06 (1.03 - 1.09)	1.05 (1.02 -1.09)	1.13 (0.99 - 1.29)	0.97 (0.95-0.99)	0.96 (0.94 -0.98)	1.02 (0.93 - 1.11)	1.15 (1.11 -1.19)	1.16 (1.12 - 1.2)	1.07 (0.92 - 1.25)
*P-trend*	*<0.001*	*<0.001*	*<0.001*	*<0.001*	*<0.001*	*0.063*	*0.001*	*<0.001*	*0.68*	*<0.001*	*<0.001*	*0.3624*
Overall Adherence												
1	1.76 (1.7 -1.82)	1.76 (1.7 - 1.82)	1.69 (1.42 - 2)	1.38 (1.26 - 1.52)	1.38 (1.25 -1.52)	1.45 (0.93 - 2.25)	0.79 (0.75-0.85)	0.70 (0.74 -0.84)	0.98 (0.73 - 1.33)	2.06 (1.84 -2.29)	2.07 (1.86 -2.32)	1.64 (0.96 - 2.79)
2	1.53 (1.49 -1.56)	1.53 (1.49 -1.57)	1.48 (1.3 - 1.68)	1.28 (1.19 - 1.37)	1.27 (1.19 -1.37)	1.32 (0.95 - 1.84)	0.84 (0.80-0.88)	0.84 (0.79 -0.88)	0.98 (0.79 - 1.24)	1.72 (1.58 -1.86)	1.73 (1.59 -1.88)	1.45 (0.97- 2.16)
3	1.33 (1.3 -1.35)	1.33 (1.31 -1.35)	1.31 (1.19 - 1.42)	1.18 (1.12 - 1.23)	1.18 (1.12 -1.23)	1.2 (0.96 - 1.5)	0.89 (0.86-0.92)	0.89 (0.86 -0.92)	0.99 (0.85 - 1.15)	1.43 (1.36 -1.51)	1.44 (1.36 -1.52)	1.28 (0.98 - 1.67)
4	1.15 (1.14 -1.16)	1.15 (1.14 -1.16)	1.14 (1.09 - 1.19)	1.08 (1.06 - 1.11)	1.08 (1.06 -1.11)	1.1 (0.98 - 1.23)	0.94 (0.93-0.96)	0.94 (0.93 -0.96)	0.99 (0.92 - 1.07)	1.2 (1.17 - 1.23)	1.2 (1.17 - 1.23)	1.13 (0.99 - 1.29)
*P-trend*	*<0.001*	*<0.001*	*<0.001*	*<0.001*	*<0.001*	*0.101*	*<0.001*	*<0.001*	*0.9207*	*<0.001*	*<0.001*	*0.06741*

All models estimated using Cox Proportional Hazards regression and adjusted for age, gender (for colorectal cancer), marital status, and education

*Abbreviations*: *CI* confidence interval, *AA* African-Americans

**Table 5:** Association (HR, 95% CI) between Adherence and Any, Breast, Colorectal and Prostate Cancer Incidence by Region, NIH-AARP Diet and Health Study

Adherence	All	Mid-West	North East	South	West
Any Cancer					
1	1.88 (1.82 - 1.95)	1.84 (1.59 - 2.14)	2.03 (1.91 - 2.17)	1.98 (1.87 - 2.10)	1.64 (1.54 - 1.75)
2	1.61 (1.57 - 1.65)	1.58 (1.41 - 1.77)	1.70 (1.63 - 1.79)	1.67 (1.60 - 1.75)	1.45 (1.38 - 1.52)
3	1.37 (1.35 - 1.4)	1.36 (1.26 - 1.46)	1.43 (1.38 - 1.47)	1.41 (1.37 - 1.45)	1.28 (1.24 - 1.32)
4	1.17 (1.16 - 1.18)	1.17 (1.12 - 1.21)	1.19 (1.18 - 1.21)	1.19 (1.17 - 1.20)	1.13 (1.11 - 1.15)
5	Ref	Ref	Ref	Ref	Ref
Breast Cancer					
1	1.44 (1.30 - 1.59)	2.03 (1.32 - 3.12)	1.53 (1.26 - 1.85)	1.36 (1.15 - 1.61)	1.38 (1.16 - 1.64)
2	1.31 (1.22 - 1.42)	1.70 (1.23 - 2.35)	1.37 (1.19 - 1.59)	1.26 (1.11 - 1.43)	1.27 (1.12 - 1.45)
3	1.20 (1.14 - 1.26)	1.42 (1.15 - 1.77)	1.24 (1.12 - 1.36)	1.17 (1.07 - 1.27)	1.17 (1.08 - 1.28)
4	1.10 (1.07 - 1.12)	1.19 (1.07 - 1.33)	1.11 (1.06 - 1.17)	1.08 (1.04 - 1.13)	1.08 (1.04 - 1.13)
5	Ref	Ref	Ref	Ref	Ref
Prostate Cancer					
1	0.77 (0.72 - 0.83)	0.82 (0.62 - 1.08)	0.77 (0.68 - 0.87)	0.79 (0.71 - 0.89)	0.75 (0.66 - 0.85)
2	0.82 (0.78 - 0.87)	0.86 (0.70 - 1.06)	0.83 (0.75 - 0.90)	0.84 (0.77 - 0.91)	0.80 (0.73 - 0.88)
3	0.88 (0.85 - 0.91)	0.91 (0.79 - 1.04)	0.88 (0.83 - 0.94)	0.89 (0.84 - 0.94)	0.86 (0.81 - 0.92)
4	0.94 (0.92 - 0.95)	0.95 (0.89 - 1.02)	0.94 (0.91 - 0.97)	0.94 (0.92 - 0.97)	0.93 (0.90 - 0.96)
5	Ref	Ref	Ref	Ref	Ref
Colorectal Cancer					
1	2.24 (2.00 - 2.52)	3.43 (2.00 - 5.88)	2.64 (2.16 - 3.24)	2.27 (1.87 - 2.75)	1.72 (1.39 - 2.14)
2	1.83 (1.68 – 2.00)	2.52 (1.68 - 3.78)	2.07 (1.78 - 2.41)	1.85 (1.60 - 2.13)	1.50 (1.28 - 1.77)
3	1.50 (1.41 - 1.59)	1.85 (1.42 - 2.42)	1.63 (1.47 - 1.80)	1.51 (1.37 - 1.66)	1.31 (1.18 - 1.46)
4	1.22 (1.19 - 1.26)	1.36 (1.19 - 1.56)	1.27 (1.21 - 1.34)	1.23 (1.17 - 1.29)	1.15 (1.09 - 1.21)
5	Ref	Ref	Ref	Ref	Ref

All models estimated using Cox Proportional Hazards regression and adjusted for age, race, gender (for any and colorectal cancer), marriage (ever, current), education (high school, college degree), and state (for all regions, and multi-state regions)

**Fig. 3:**
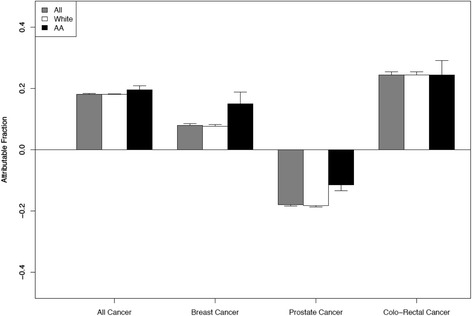
Attributable fraction (%, 95% CI) for adherence by race and cancer type

The proportion of cancer incidence attributable to low adherence was higher among African-Americans compared with Whites for all cancers (21% vs. 19%), and highest for colorectal cancer (25%) regardless of race. Racial difference in the attributable fraction was observed for breast and prostate cancer: 16% of breast cancer incidence was attributable to low adherence for African-American and less than 8% for Whites. Notably, 18% of prostate cancer incidence was prevented due to low adherence overall; 12% for African-American and 18% for Whites (Fig. 3).

## Discussion

In one of the largest prospective cohort studies of older adults in the US, we observed racial, gender and regional differences in the level of adherence to AICR/WCRF cancer-related risk factor guidelines. At baseline, adherence was overwhelmingly low, with less than 2% of older adults adherent to all five criteria; less than 1% of African-American and 1.5% of Whites met all five criteria for body weight, physical activity, smoking, alcohol and diet. Adherence was highest in the West for obesity and physical activity, and in the North East for alcohol use, smoking and nutrition. Cancer risk overall increased significantly with reduced adherence to the cancer-related risk factor guidelines; adherence to one or fewer criteria (relative to five) increased the risk of all cancers by 76%, breast cancer by 38%, and colorectal cancer by 100%, however lower adherence was associated with a 21% reduced risk of prostate cancer. Although the magnitude of the associations was similar between African-American and Whites, the only statistically significant association for African-Americans was for the risk of any cancer and not for specific cancers. Overall, lower adherence was associated with increased cancer risk consistently across regions, except for colorectal cancer where there was a higher but non-significant association in the Mid-West. About 20% of all cancers, 10% of breast and 24% of colorectal cancers are attributable to low adherence, however among White women, only 8% of breast cancer incidence was attributable to low adherence, compared with 18% for African-American women, and close to 20% of prostate cancer cases were actually prevented by low adherence.

Several studies have examined the influence of cancer-related risk factors in general, and adherence to cancer prevention guidelines, on the risk of developing cancer and have observed similar results to ours [19–22]. However, no other study has examined race-gender-region differences in the level of adherence among older adults, and assessed whether the association with cancer incidence was similar across racial groups. This gap has been a major limitation in the previous literature for many reasons. First, given the progressively ageing population of the US [23], the influence of modifiable lifestyle risk factors on cancer risk deserves more attention that it has received. For the most common cancers, especially breast, prostate and colorectal, there is no single etiologic risk factor that explains the risk of cancer development beyond age and lifestyle related modifiable factors such as obesity, diet, physical activity, smoking and alcohol [24]. We find that the attributable risk due to these lifestyle risk factors is close to 20%, i.e. about 20% of new cancer cases could have been prevented due to complete adherence. Second, the highly aggressive and fast growing nature of tumors prevalent among African-Americans suggests that there may be certain uniquely-patterned risk factors in this population group that may only be identified with population-specific studies [25]. Third, if cancer prevention strategies are developed focusing on specific risk factors and targeted to race-gender-region population sub-groups where they are most needed [26], with considerations of unique facilitators and barriers to adherence in those sub-groups, they may be more likely to succeed compared with one-size fits all approaches to cancer prevention [27].

The biological mechanisms linking modifiable lifestyle factors and cancer development have been well established, including in a comprehensive review by [28]. Excess calorie intake and low physical activity are associated with increased accumulation of adipose tissue, leading to overweight and obesity [29]. These in turn lead to hyperglycemia, hypertriglyceridemia, inflammation and insulin resistance [30], which have been shown to increase the risk of breast and colorectal cancer incidence, as well as the development of the more aggressive hormone-receptor negative sub-types of breast cancer [31, 32]. Other pathways include the alteration of circulating adipokines, altered secretion of sex hormones such as estrogen and androgen, as well as multiple inflammatory markers such as cytokines [33]. While moderate alcohol intake has been associated with reduced risk for some types of cardiovascular diseases [34], the association in cancer has been most studied in relation to breast cancer, with results suggesting a modest increase in incidence associated with higher alcohol consumption [35]. We observed that higher alcohol use was associated with significantly increased risk of cancers in both racial groups, however stronger associations were observed among African-Americans compared with Whites. African-Americans with excess alcohol use were at more than a 100% increased risk of breast cancer, and almost 300% increased risk of colorectal cancer compared with a 50% increased risk of breast cancer and 100% increased risk of colorectal cancer. The biological mechanism linking this association may involve race-specific differences in alcohol metabolism, alterations in inflammatory response and/or interactions with underlying comorbid conditions. Non-biological mechanisms such as differences in the type of alcohol consumed (e.g. wine, beer, spirits) or drinking patterns (e.g. binge drinking) may also play a role.

Genetic and epigenetic alterations in cancer-related genes, influenced by lifestyle factors, have also been shown to influence cancer tumorigenesis [36]. Nevertheless, our observation of racial differences in the proportion of breast and prostate cancer cases attributable to adherence suggests that the same risk factor may exert more severe biological effects on certain racial groups compared with others, and research studies focused on identifying the mechanisms underlying these differences, for example due to biological interactions or synergy between cancer-related risk factors and underlying comorbidities, may provide information on the causal components for these major cancer types.

Despite convincing evidence regarding the negative influence of obesity, smoking, and low physical activity on health outcomes in general, and cancer risk specifically, we observed that in 1995–1996 only about a third of older US adults met each of the modifiable lifestyle risk factors (except for alcohol use) [37]. These estimates have remained consistent based on recent 2014 BRFSS data showing that 65% of US adults were overweight/obese, 77% consumed less than five servings of fruits and vegetables per day, 49% did not engage in adequate physical activity, and 18% were current smokers. The lower levels of adherence to the risk factors observed among African-Americans compared with Whites suggests that socio-economic differences may play a major role [20, 38–40]. Multiple studies have observed significant associations between socio-economic status and increased risk of cancer [38]. Our results suggest that a possible conceptual pathway for racial disparities in cancer risk would involve race influencing socio-economic status, which in turn influences cancer risk through adherence to cancer related risk factors [40–43]. Thus, a realistic strategy to preventing cancer risk and reducing racial disparities in cancer could involve population specific public health strategies to improve adherence to these common risk factors. For instance, improving access to low-cost fresh fruits and vegetables in low-income communities of the US in general, and the South in particular given that only 24% of Southern adults in this study consumed recommended servings of fruits and vegetables; improving public safety and neighborhood walkability to encourage recreational physical activity especially in the Mid-West given that only 18% of Mid-Western adults in this study met recommended physical activity levels; better understanding of culture-specific tobacco cessation programs that are most likely to be effective, especially in the South where only 34% of adults in this study were non-smokers.

We observed an inverse association between adherence and prostate cancer risk. This is similar to findings from other studies [15, 44, 45], as well as an updated WCRF report [46] showing null or inverse associations between lifestyle risk factors except a probable association between body weight and prostate cancer. The association between smoking and prostate cancer may be due to potential detection bias, since smokers may be less health conscious and less likely to be diagnosed with cancer, or a yet unidentified genetic or molecular risk factor. The observed inverse association may also be due to competing risks; since prostate cancer is a slow, indolent cancer type, individuals at lower levels of adherence may die earlier due to other lifestyle associated factors e.g. cardiovascular diseases prior to prostate cancer diagnosis. Nevertheless, prostate cancer remains one of the most common cancers among men in the US, with markedly higher risk and aggressiveness among African-American men compared with Whites. Further research studies will be needed to identify etiological factors that may be modifiable to inform prostate cancer prevention efforts. The current analysis is strengthened by the availability of large sample sizes for both African-Americans and Whites, a long duration of follow-up and lower likelihood of recall bias, and comprehensive set of study covariates for confounder adjustment. There were also a few limitations to this study. First, since NIH-AARP was a large cohort study of health status of older adults in general, there was less detailed information on some cancer-specific risk factors such as frequency of cancer screening such as mammography or PSA screening. Second, self-reported dietary patterns may be vulnerable to measurement error and may have led to an underestimation of the association with cancer risk, and examination of fruit and vegetable intake alone may have obscured race-specific dietary patterns that may be important for cancer risk. Finally, risk factors were assessed at baseline, however there is considerable interest in identifying the etiologic window over the entire lifecourse at which adherence is most important, i.e. early life, early adulthood or in older ages, which may further inform efforts to better target cancer prevention messages.

## Conclusion

In conclusion, for the major cancer types observed among US adults, lack of adherence to lifestyle related cancer risk factor guidelines significantly increased cancer risk, with up to 25% of new cancer cases attributable to low adherence. A larger proportion of breast cancer incidence in African-American women compared with Whites was attributable to examined lifestyle related risk factors, suggesting that there may be unique opportunities for targeted clinical and public health strategies to reduce the burden of breast cancer among older African-American adults.

## Additional file

Additional file 1: Participant flowchart for NIH_AARP Diet and Health Study. The flow chart shows how many participants were in the cohort from start to finish. (PPTX 63 kb)

## Abbreviations

ACS: American Cancer Society
AICR: American Institute for Cancer Research
AR: Attributable risk
HR: Hazard ratio
NIH-AARP: National Institutes of Health-American Association of Retired Persons
PSA: Prostate specific antigen
RR: Risk ratio
WCRF: World Cancer Research Fund
