# Supplementary material for: Cancer-related risk factors and incidence of major cancers by race, gender and region; analysis of the NIH-AARP diet and health study
Source: BMC Cancer. 2017 Aug 30;17:597. doi: 10.1186/s12885-017-3557-1 (PMC5577755; doi:10.1186/s12885-017-3557-1)
Supplement: Additional file 1: — Participant flowchart for NIH_AARP Diet and Health Study. The flow chart shows how many participants were in the cohort from start to finish. (PPTX 63 kb) [file 12885_2017_3557_MOESM1_ESM.pptx]

## Slide 1
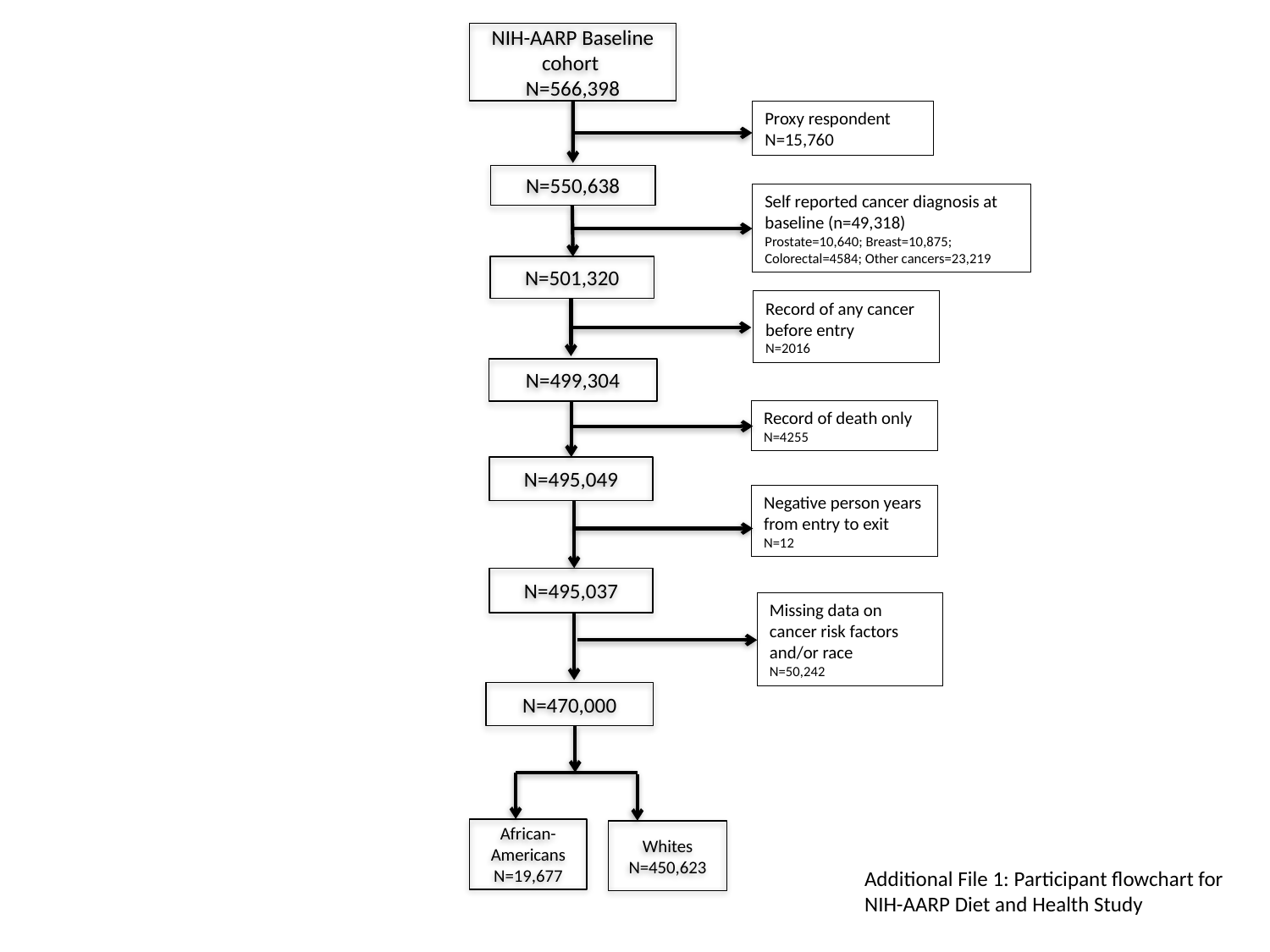

NIH-AARP Baseline cohort
N=566,398
Proxy respondent
N=15,760
N=550,638
Self reported cancer diagnosis at baseline (n=49,318)
Prostate=10,640; Breast=10,875; Colorectal=4584; Other cancers=23,219
N=501,320
Record of any cancer before entry
N=2016
N=499,304
Record of death only
N=4255
N=495,049
Negative person years from entry to exit
N=12
N=495,037
Missing data on cancer risk factors and/or race
N=50,242
N=470,000
African-Americans N=19,677
Whites
N=450,623
Additional File 1: Participant flowchart for NIH-AARP Diet and Health Study
